# *γ*-Glutamylcysteine Alleviates Ischemic Stroke-Induced Neuronal Apoptosis by Inhibiting ROS-Mediated Endoplasmic Reticulum Stress

**DOI:** 10.1155/2021/2961079

**Published:** 2021-11-16

**Authors:** Hui-qin Li, Sheng-nan Xia, Si-yi Xu, Pin-yi Liu, Yue Gu, Xin-yu Bao, Yun Xu, Xiang Cao

**Affiliations:** ^1^Department of Neurology, Drum Tower Hospital, Medical School and The State Key Laboratory of Pharmaceutical Biotechnology, Institute of Brain Science, Nanjing University, Nanjing 210008, China; ^2^Jiangsu Key Laboratory for Molecular Medicine, Medical School of Nanjing University, Nanjing 210008, China; ^3^Jiangsu Province Stroke Center for Diagnosis and Therapy, Nanjing 210008, China

## Abstract

Ischemic stroke is a severe and acute neurological disorder with limited therapeutic strategies currently available. Oxidative stress is one of the critical pathological factors in ischemia/reperfusion injury, and high levels of reactive oxygen species (ROS) may drive neuronal apoptosis. Rescuing neurons in the penumbra is a potential way to recover from ischemic stroke. Endogenous levels of the potent ROS quencher glutathione (GSH) decrease significantly after cerebral ischemia. Here, we aimed to investigate the neuroprotective effects of *γ*-glutamylcysteine (*γ*-GC), an immediate precursor of GSH, on neuronal apoptosis and brain injury during ischemic stroke. Middle cerebral artery occlusion (MCAO) and oxygen-glucose deprivation/reoxygenation (OGD/R) were used to mimic cerebral ischemia in mice, neuronal cell lines, and primary neurons. Our data indicated that exogenous *γ*-GC treatment mitigated oxidative stress, as indicated by upregulated GSH and decreased ROS levels. In addition, *γ*-GC attenuated ischemia/reperfusion-induced neuronal apoptosis and brain injury in vivo and in vitro. Furthermore, transcriptomics approaches and subsequent validation studies revealed that *γ*-GC attenuated penumbra neuronal apoptosis by inhibiting the activation of protein kinase R-like endoplasmic reticulum kinase (PERK) and inositol-requiring enzyme 1*α* (IRE1*α*) in the endoplasmic reticulum (ER) stress signaling pathway in OGD/R-treated cells and ischemic brain tissues. To the best of our knowledge, this study is the first to report that *γ*-GC attenuates ischemia-induced neuronal apoptosis by suppressing ROS-mediated ER stress. *γ*-GC may be a promising therapeutic agent for ischemic stroke.

## 1. Introduction

Stroke has become the second leading cause of death worldwide and seriously affects public health. Statistically, ischemic stroke accounts for approximately 80% of all strokes [1]. Although recanalization treatment with recombinant tissue plasminogen activator (Rt-PA) is effective, the benefiting population is less than 10% due to the time window problem [2]. In fact, reperfusion injury mediated by recanalization often occurs. Therefore, there is a desperate need for the development of new therapeutic strategies to prevent ischemic brain injury.

Most researchers now agree that neurons in the ischemic penumbra are in a state of electrical failure; although neuronal function is abnormal, these cells are still viable. Improvements in ischemic penumbra neuronal state and function directly influence the outcome of stroke [3, 4]. Although the pathophysiological mechanisms of reperfusion injury are complex, it has been demonstrated that excessive generation of reactive oxygen species (ROS) is a hallmark of reperfusion injury [5, 6]. During the acute phase of cerebral ischemia, blood vessel blockade leads to calcium overload and energy depletion in nerve cells, which can induce the accumulation of ROS and subsequent brain injury after the resupply of oxygen and nutrients. Excessive formation of ROS caused by ischemic stroke overwhelms the endogenous antioxidant system and induces neuronal death via multiple pathways, including endoplasmic reticulum (ER) stress-induced apoptosis and ferroptosis [7, 8]. Thus, attenuating excessive ROS production can reduce the apoptosis of penumbra neurons and protect against ischemic brain injury.

Accumulating evidence has shown that ROS cannot be readily scavenged because of the low activities and levels of antioxidants in the ischemic brain [9, 10]. Edaravone is a currently listed drug used to treat ischemic stroke in the clinic partly by capturing and reducing excessive ROS [10, 11]. However, renal disorders that occur during or after treatment with edaravone have drawn attention [12, 13]. In addition to medications, some endogenous substances can also scavenge ROS. Among them, glutathione (GSH) is a potent quencher of ROS [14]. It has been suggested that GSH levels are greatly reduced in the cortex and striatum of the infarcted hemisphere. Oral administration of exogenous GSH exerts a protective effect on rats with ischemic stroke [15]. N-Acetylcysteine (NAC) is a GSH precursor that has been reported to increase neuronal survival and reduce infarct volume in rat ischemia models of middle cerebral artery occlusion (MCAO) [16]. Although animal model studies have shown that exogenous GSH and NAC have therapeutic effects on cerebral ischemia, these agents are still far from ideal for clinical applications. Exogenous GSH can be rapidly metabolized in the blood, and the delivery of GSH to the central nervous system is hampered by the presence of the blood brain barrier (BBB) [17]. For NAC, three enzymes are involved in the synthesis of GSH from NAC, and *γ*-glutamylcysteine ligase (GCL) is a rate-limiting enzyme of this process [18]. Moreover, the poor oral bioavailability of NAC also hampers its clinical use [19].

*γ*-Glutamylcysteine (*γ*-GC) is an immediate precursor of GSH. Unlike NAC, *γ*-GC was suggested to promote GSH synthesis and only requires catalysis by glutathione synthetase (GSS), and this way of synthesizing GSH is easier and simpler [20, 21]. Moreover, exogenous *γ*-GC can readily cross the BBB and be taken up by many cell types. Liu et al. found that dietary supplementation with *γ*-GC improved the spatial memory of Alzheimer's disease (AD) mice by increasing total GSH and reducing the levels of oxidative stress and nerve cell apoptosis [22]. A recent study showed that *γ*-GC exerts antioxidative and anti-inflammatory effects against oligomeric A*β*_40_-induced astrocyte injury [23]. Additionally, *γ*-GC has been shown to inhibit oxidative injury in endothelial cells [24]. Despite these findings, much less is known about the effects of *γ*-GC on ROS-mediated neuronal apoptosis after ischemic stroke.

In this study, we examined the neuroprotective role of *γ*-GC in the HT22 neuronal cell line and primary cortical neurons exposed to oxygen-glucose deprivation/reoxygenation (OGD/R) injury and in mice subjected to MCAO-induced ischemia-reperfusion injury. Furthermore, we conducted transcriptomics analysis to explore the potential molecular and cellular pathways underlying these protective effects of *γ*-GC. These results provide a novel therapeutic strategy for treating cerebral ischemia.

## 2. Methods

### 2.1. Reagents and Antibodies

*γ*-GC with a purity of over 95% (CAS no. 636-58-8) was purchased from Shanghai Yuanye Bio-Technology Co., Ltd. (Shanghai, China). Antibodies against 78 kDa glucose-regulated protein (GRP78), protein kinase R-like endoplasmic reticulum kinase (PERK), inositol-requiring enzyme 1*α* (IRE1*α*), TNF receptor-associated factor 2 (TRAF2), eukaryotic initiation factor 2 (eIF2*α*), phosphorylated eIF2*α* (p-eIF2*α*), C/EBP homologous protein (CHOP), c-Jun NH 2-terminal kinase (JNK), and phosphorylated JNK (p-JNK) were obtained from Cell Signaling Technology (Massachusetts, USA). Antibodies against Bax, Bcl-2, and GAPDH were purchased from Bioworld Technology (St. Louis, Missouri, USA). Antibodies against caspase-3 were purchased from Proteintech Group (Wuhan, China). An antibody against p-PERK was obtained from Signalway Antibody LLC (Maryland, USA), and an antibody against p-IRE1*α* was obtained from Affinity Biosciences (Jiangsu, China). Cell Counting Kit-8 (CCK-8) and calcein-AM/propidium iodide (PI) were purchased from Dojindo Molecular Technologies (Tokyo, Japan). An Annexin V-PE/7-AAD apoptosis detection kit and TUNEL kit were obtained from Vazyme Biotech Co. (Nanjing, China). ROS, malondialdehyde (MDA), and GSH assay kits were obtained from Beyotime Biotechnology (Shanghai, China).

### 2.2. Cell Culture and Animals

HT22 neuronal cells were maintained in DMEM supplemented with 10% FBS, penicillin, and streptomycin and incubated at 37°C under 5% CO_2_. Cells were subcultured at an interval of 2 days. Primary cortical neurons were dissociated from E15-17 C57/BL6J mouse embryos as previously described [25]. The isolated cortical neurons were seeded on poly-D-lysine-coated plates and cultured in neurobasal-A media containing B27 and glutamine. Experiments were initiated on culture days 7-8.

Seven- to eight-week-old male C57/BL6J mice were purchased from the Animal Model Center of Nanjing Medical University (Nanjing, Jiangsu, China). All mice were housed in specific pathogen-free (SPF) conditions and allowed ad libitum access to food and water. Experimental operations on mice were performed according to the Guide for the Animal Care and Use Committee of Nanjing University.

### 2.3. OGD/R Insult

OGD/R was performed on HT22 cells and primary cortical neurons as previously described with minor modifications [25]. Cells were placed in serum/glucose-free medium and then transferred to an anaerobic chamber flushed with a mixture of 5% CO_2_ and 95% N_2_ at 37°C. Then, the cultures were switched to the original conditions, and the cells were returned to an aerobic environment. The exposure times for HT22 cells and primary cortical neurons to OGD insult were 40 min and 12 h, respectively. The reoxygenation time was modified in different experiments.

### 2.4. Focal Cerebral Ischemia and Laser Speckle Contrast Imaging (LSCI)

Mice were randomly allotted to five groups: sham, MCAO+solvent, MCAO+300 mg/kg *γ*-GC, MCAO+600 mg/kg *γ*-GC, and MCAO+900 mg/kg *γ*-GC. Except for mice in the sham group, all mice were subjected to MCAO to establish a transient focal cerebral ischemia model. In brief, mice were deeply anesthetized with an intraperitoneal injection of pentobarbital sodium. Next, after dissecting the right common carotid artery, external carotid artery, and internal carotid artery, a suture (Doccol Corporation, MA, USA) was inserted via the external carotid artery into the internal carotid artery until a steep drop in regional cerebral flow was observed. After 1 h, the suture was removed to establish reperfusion. Fifteen minutes from the onset of reperfusion, the mice were intragastrically administered *γ*-GC or saline. During the surgery, body temperature was maintained at 37 ± 0.5°C with a heating pad. Sham-operated mice were subjected to all procedures except for insertion of the suture.

To ensure the success of the MCAO model, LSCI was used to visualize cerebral blood flow. The surface of the mouse skull was exposed to a laser (Perimed Corporation, Stockholm, Sweden), and regions of interest were then selected to assess the speckle contrast over time. Images were captured to further analyze the changes in cerebral blood flow.

### 2.5. Cell Viability Assay

The CCK-8 assay was used to determine the viability of HT22 cells and primary cortical neurons. Cells were plated in 96-well plates and subjected to OGD/R. At the end of reoxygenation, 10 *μ*l of CCK-8 was added per well and incubated at 37°C. Finally, the optical density (OD) was measured at 450 nm with a microplate reader (Tecan Trading AG, Switzerland).

### 2.6. Flow Cytometric Apoptosis Assays

HT22 cells were stained with Annexin V and 7-AAD and then analyzed by flow cytometry to quantify apoptosis. Briefly, after OGD/R insult, both suspension and adherent HT22 cells were harvested and washed with cold phosphate-buffered saline (PBS). Next, the cells were resuspended in a working solution containing Annexin V-PE and 7-AAD and incubated at room temperature for 15 min in the dark. HT22 cells were immediately analyzed by flow cytometry. More than 1 × 10^4^ cells were recorded in each sample, and the experiment was repeated 3 times.

### 2.7. Calcein and PI Dual Labeling

Primary cortical neuronal apoptosis was evaluated with the fluorescent dyes calcein and PI. The calcein-labeled neurons exhibiting green fluorescence were viable, while PI-labeled neurons with red fluorescence were apoptotic. After OGD/R insult, primary cortical neurons were incubated with calcein-AM and PI for 15 min at 37°C in the dark. After being washed, the neurons were analyzed by using a fluorescence camera, and images were captured to identify live/apoptotic neurons (Olympus BX51, Japan).

### 2.8. TUNEL Assay

In situ apoptosis was evaluated by the TUNEL assay according to the manufacturer's instructions. Briefly, 24 h after the MCAO operation, the mice were subjected to transcardial perfusion with 0.9% saline and 4% paraformaldehyde (PFA) in sequence under deep anesthesia. After being fixed and dehydrated, the frozen brains were sliced into 20 *μ*m sections for subsequent experiments. After fixation and permeabilization, the brain slices were incubated with the TUNEL assay mixture for 2 h. The nuclei were stained with DAPI. The TUNEL-labeled cells were visualized with a fluorescence microscope.

### 2.9. Detection of ROS

The generation of ROS was measured by using the cell permeant reagent 2′,7′-dichlorofluorescin diacetate (DCFH-DA). After being subjected to OGD/R and MCAO, the adherent cells and brain slices, respectively, were washed and then stained with DCFH-DA in the dark. Next, the samples were washed three times to remove extracellular DCFH-DA. Finally, intracellular DCFH was oxidized by ROS into 2′,7′-dichlorofluorescein (DCF), which was detected by fluorescence spectroscopy at excitation/emission wavelengths of 488 nm/525 nm, indicating the level of ROS.

### 2.10. Detection of MDA

MDA is a biomarker of lipid peroxidation, which is an important component of oxidative stress. The level of MDA was measured according to the manufacturer's instructions. Briefly, the harvested brain tissue was homogenized in cold PBS and centrifuged at 12,000 g for 10 min to collect the supernatant. After the MDA detection working solution was added, all standards and samples were incubated at 100°C for 15 min. The absorbance was immediately measured at a wavelength of 532 nm.

### 2.11. Measurement of Total GSH

The level of total GSH was determined using a commercial kit according to the manufacturer's instructions. The harvested specimens were prepared according to the protocol and then incubated with the prepared working solution. The absorbance was measured at 412 nm with a microplate reader.

### 2.12. RNA Sequencing and Differentially Expressed Gene (DEG) Analysis

Total RNA was extracted from HT22 cells using the TRIzol reagent. Quality assessment was determined by using a NanoDrop 2000 (Thermo Scientific, USA) and Agilent 2100 Bioanalyzer (Agilent, Santa Clara, CA, USA). Transcriptome sequencing and analysis were conducted by OE Biotech Co., Ltd. (Shanghai, China). Hierarchical cluster analysis of DEGs was carried out to identify the expression patterns of genes in different groups. GO enrichment and KEGG pathway enrichment analyses were performed to further analyze the DEGs.

### 2.13. Behavioral Testing

The neurological deficits of MCAO mice were evaluated 24 h postoperation by behavioral testing, including the modified neurological severity score (mNSS), grip strength, and rotarod test. The mNSS was used to assess motor, sensory, reflex, and balance deficits. The score ranges from 0 to 18, and a higher score indicates more severe deficits. Grip strength was measured with a grip strength meter (GS3, Bioseb, France). Mouse forepaws were allowed to grasp the platform of the strength meter five times, and the maximum value was recorded. Prior to MCAO, the mice were trained for 3 days on rotarods (RWD Life Science, Shenzhen, China). 24 h after MCAO, the mice were placed on the rotarod, which was accelerated linearly from 0 to 40 rpm/min. The time the mice struggled on the rotarod was recorded, and the maximum time was 300 s.

### 2.14. Infarct Volume Evaluation

The infarct volume was measured by staining with 2% 2,3,5-triphenyltetrazolium chloride (TTC, Sigma-Aldrich). After behavioral testing, the mice were euthanized, and the brains were sliced into 2 mm sections. Then, the brain slices were incubated with 2% TTC at 37°C for 20 min. The infarct area remained unstained, while the normal region was stained red. The subsequent analysis was conducted by using ImageJ software (ImageJ 1.5, NIH, USA), and the percentage of hemispheric infarction volume was calculated as follows: (contralateral hemisphere area–noninfarct ipsilateral hemisphere area)/(2∗contralateral hemisphere area)∗100%.

### 2.15. Western Blot Analysis

Samples were homogenized in RIPA buffer containing a protease inhibitor cocktail to extract total proteins. The proteins were separated by SDS-PAGE and then transferred onto PVDF membranes. Then, the PVDF membranes were blocked with blocking buffer for 60 min at room temperature and incubated with the indicated primary antibodies overnight at 4°C. Subsequently, membranes were incubated with HRP-conjugated secondary antibodies. Protein bands were visualized by using an ECL Detection Kit, and images were captured by a Gel-Pro system (Tanon Technologies, Shanghai, China). The band intensities were quantified using ImageJ software.

### 2.16. Immunoprecipitation

After OGD/R, HT22 cells were lysed in RIPA buffer in the presence of protease inhibitors. After centrifugation, the supernatant was incubated with an antibody against IRE1*α* overnight at 4°C. Subsequently, precleared Protein A/G PLUS-agarose beads (Millipore, Billerica, MA, USA) were added to the mixture to bind the immune complexes at 4°C for 3 h. Finally, the eluted antigens were subjected to SDS-PAGE.

### 2.17. Immunofluorescence Staining

The brain slices in the sham, MCAO+solvent, and MCAO+*γ*-GC groups were dehydrated at 37°C for 30 min. Next, the sections were permeabilized with 0.2% Triton X-100 and blocked with 2% bovine serum albumin (BSA). Brain slices were incubated with primary antibodies against p-IRE1*α*, p-eIF2*α*, and NEUN overnight at 4°C. The next day, the brain sections were incubated with the corresponding secondary antibodies, and the nuclei were stained with DAPI. Images were captured using confocal fluorescence microscopy (Olympus FV3000, Japan).

### 2.18. Statistical Analysis

The data in this study were normally distributed and are presented as the mean ± standard error of the mean (SEM). Comparisons between groups were determined by one-way ANOVA followed by Tukey's multiple comparison tests, and cerebral blood flow was analyzed by two-way ANOVA. A *p* value less than 0.05 was considered significantly different. All data were analyzed by using SPSS 18.0 software.

## 3. Results

### 3.1. *γ*-GC Protects HT22 Neurons against OGD/R-Induced Death

**Figure 1:**
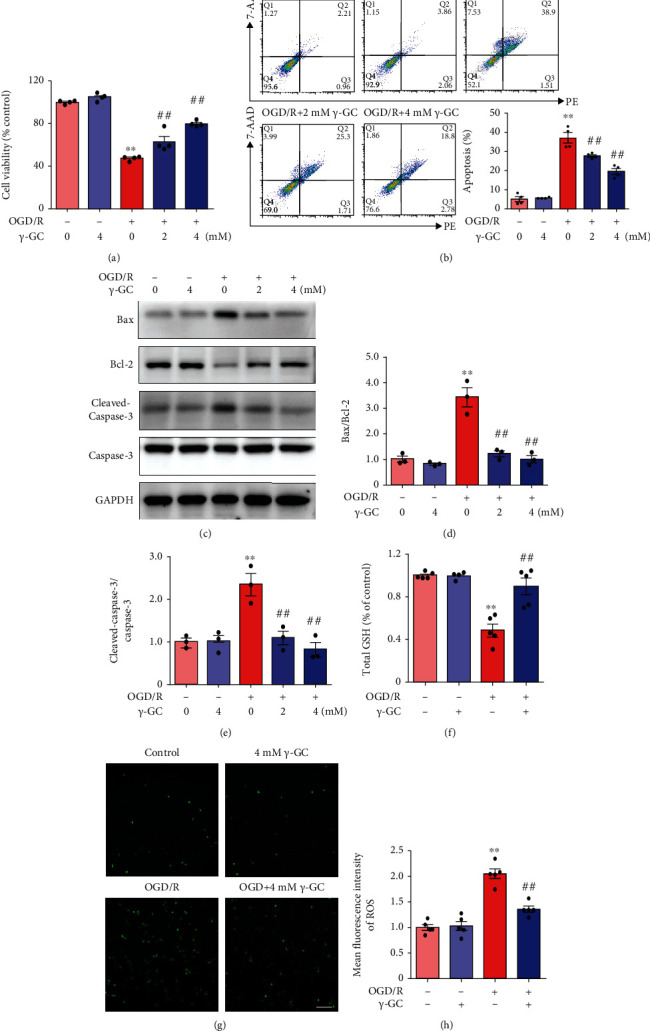
*γ*-GC reduced apoptosis and oxidative injury in HT22 cells induced by OGD/R. HT22 cells were pretreated with *γ*-GC for one hour prior to OGD and then exposed to OGD for 12 h, followed by 12 h of reperfusion. *γ*-GC was present throughout the entire process of OGD and reperfusion. (a) Cell viability was measured by the CCK-8 assay. (b) HT22 cell apoptosis was assessed by Annexin V/7-AAD staining. Annexin V^+7^-AAD^−^ and Annexin V^+7^-AAD^+^ cells were defined as apoptotic cells. (c) The ratios of Bax/Bcl-2 and cleaved caspase-3/caspase-3 were measured by western blot analysis after 6 h of reperfusion. (d, e) Quantification of the levels of Bax/Bcl-2 and cleaved caspase-3/caspase-3. In the following experiments, HT22 cells were subjected to 12 h of reperfusion. (f) Quantification of total GSH. (g, h) The level of intracellular ROS. Scale bar: 100 *μ*m. All data are presented as the mean ± SEM. ^∗^*p* < 0.05, ^∗∗^*p* < 0.01 versus the control group. ^**#**^*p* < 0.05, ^**##**^*p* < 0.01 versus the OGD/R group.

To determine whether *γ*-GC can alleviate neuronal injury induced by ischemia/reperfusion, we first explored the effects of *γ*-GC treatment on HT22 cells exposed to OGD/R. The percent cell viability detected by the CCK-8 assay was significantly decreased to 47.06 ± 1.07% by OGD/R, while the addition of 2 mM and 4 mM *γ*-GC increased cell viability to 62.83 ± 4.93% and 79.66 ± 1.39%, respectively (Figure 1(a)). The results also revealed that up to 4 mM *γ*-GC exerted no cytotoxic effect on HT22 cells. Moreover, HT22 cells treated with *γ*-GC showed decreased apoptosis rates (Figure 1(b)). After OGD/R insult, an increased percentage of HT22 cells were labeled with Annexin V and 7-AAD; 2 mM and 4 mM *γ*-GC reversed this increase to some extent, with 4 mM *γ*-GC having a superior effect. To further determine the antiapoptotic effect of *γ*-GC on the HT22 OGD/R model, Bax, Bcl-2, and cleaved caspase-3 were measured by western blot analysis. As shown in Figures 1(c)–1(e), OGD/R increased the ratio of Bax/Bcl-2 and the level of cleaved caspase-3. HT22 cells treated with *γ*-GC exhibited marked reductions in the level of cleaved caspase-3 and the ratio of Bax/Bcl-2. Based on these results, 4 mM *γ*-GC was selected as an appropriate concentration for subsequent experiments. Given the role of *γ*-GC in the process of GSH synthesis, we further measured the total GSH level and intracellular ROS level and found that compared to the OGD/R group, the 4 mM *γ*-GC group exhibited increased levels of total GSH and high intracellular ROS scavenging capacity (Figures 1(f)–1(h)). Overall, *γ*-GC displayed neuroprotective effects in the HT22 OGD/R model, and the underlying mechanism awaited further study.

### 3.2. Transcriptome Analysis of HT22 Cells after OGD/R Challenge

**Figure 2:**
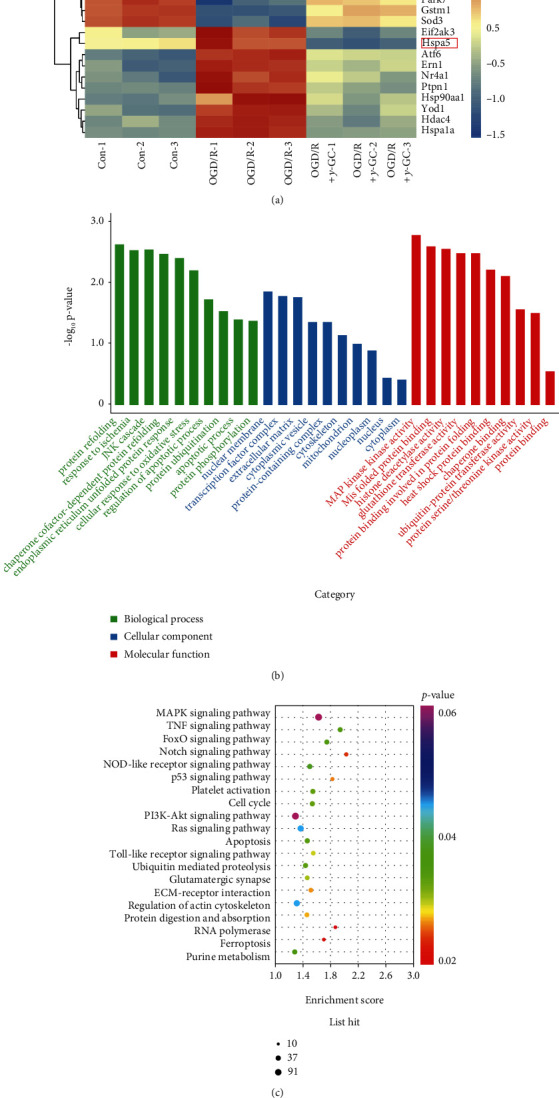
The DEGs of HT22 cells exposed to 12 h of OGD and 3 h of reperfusion were analyzed by transcriptome sequencing. (a) Heatmap of DEGs between the control group, OGD/R group, and OGD/R+*γ*-GC group (4 mM). (b) List of enriched GO analyses of biological processes, cellular components, and molecular functions. (c) Scatter plot of KEGG pathway enrichment analysis of DEGs.

To identify the potential mechanism by which *γ*-GC protects HT22 cells against OGD/R insult, we performed RNA-seq on HT22 cells in the control group, the OGD/R group, and the OGD/R+*γ*-GC group, and 17731 genes were identified. Based on the fold change (≥1.5) and *p* value (≤0.05), DEG comparisons were conducted between the three groups. Compared to the control group, HT22 cells in the OGD/R group had 1934 upregulated and 1387 downregulated DEGs. Compared to HT22 cells in the OGD/R group, those in the OGD/R+*γ*-GC group had 1131 upregulated and 1697 downregulated DEGs. Focusing on the DEGs that were downregulated in the OGD/R group relative to the control group but upregulated in the OGD/R+*γ*-GC group relative to the OGD/R group, as well as those that were upregulated in the OGD/R group relative to the control group but downregulated in the OGD/R+*γ*-GC group relative to the OGD group, we further analyzed the changes in gene expression. As shown in Figure 2(a), the significant DEGs Bcl-2, Mapk3, and caspase-12 were associated with apoptosis and proliferation. In addition, Oxr1, Prdx5, and Mgst1 are members of the antioxidant system, and Eif2ak3, Hspa5, Atf6, and Ern1 play important roles in ER stress. In particular, Hspa5 encodes GRP78, an essential regulator of the unfolded protein response (UPR) pathway. Furthermore, GO analysis and KEGG pathway analysis (Figures 2(b) and 2(c)) indicated that *γ*-GC may play a role in neuroprotection by attenuating apoptosis, ameliorating oxidative injury and alleviating ER stress, which is in accordance with the heatmap analysis.

### 3.3. *γ*-GC Inhibits ER Stress-Induced Apoptosis in HT22 Cells

**Figure 3:**
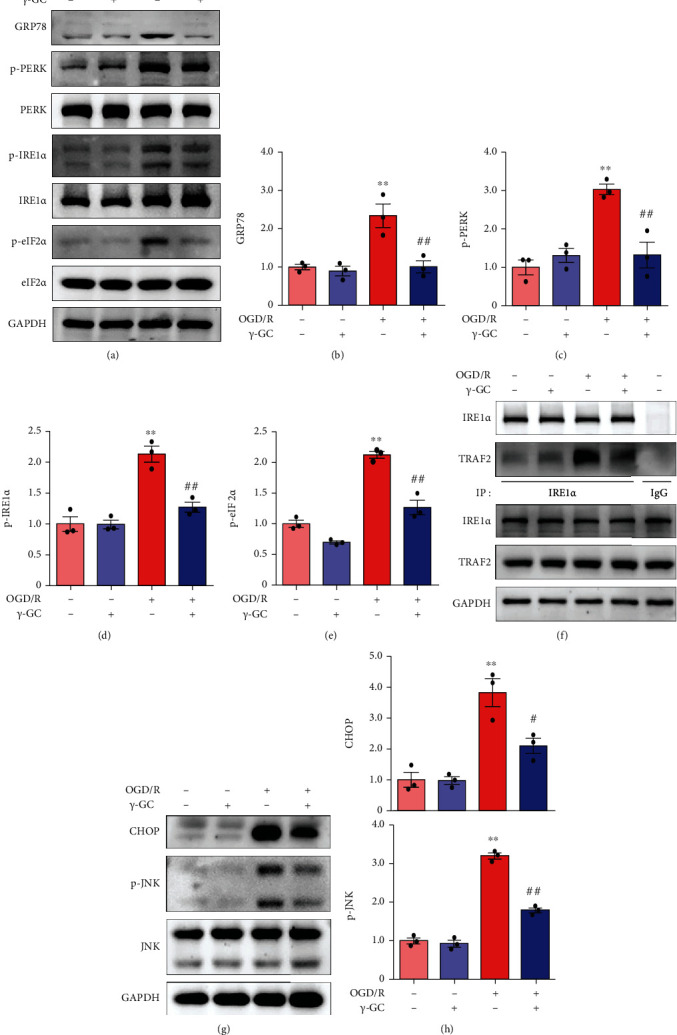
*γ*-GC protected HT22 cells against OGD/R insult by repressing ER stress-induced apoptosis. After HT22 cells were subjected to 12 h of OGD and 1 h of reperfusion, the following experiments were conducted. (a) Western blot analysis of ER stress-associated proteins, including GRP78, p-PERK, p-IRE1*α*, and p-eIF2*α*. (b–e) Quantification of the levels of GRP78, p-PERK/PERK, p-IRE1*α*/IRE1*α*, and p-eIF2*α*/eIF2*α*. (f) The interaction between IRE1*α* and TRAF2 was evaluated by immunoprecipitation. (g) CHOP and p-JNK, which are indicators of ER-induced stress, were analyzed by western blotting at 6 h postreperfusion. (h) Quantification of CHOP and p-JNK/JNK expression. All data are presented as the mean ± SEM. ^∗^*p* < 0.05, ^∗∗^*p* < 0.01 versus the control group. ^**#**^*p* < 0.05, ^**##**^*p* < 0.01 versus the OGD/R group.

According to the RNA-seq analysis, the mechanism of *γ*-GC neuroprotection is likely to be associated with relieving oxidative and ER stress. It has been shown that excessive ROS are the predominant contributors to ER stress and exacerbate ER-induced apoptosis [26]. Then, we verified whether *γ*-GC inhibited ER stress-induced apoptosis by western blot analysis and immunoprecipitation. OGD/R markedly increased the level of GRP78 in HT22 cells (Figures 3(a) and 3(b)), suggesting a burst of ER stress. *γ*-GC alleviated ER stress, as indicated by the decreased level of GRP78. Moreover, western blot analysis clearly revealed that *γ*-GC reduced the activation of two downstream factors, PERK and IRE1*α*, which were activated by OGD/R insult (Figures 3(a), 3(c), and 3(d)). The autophosphorylation of PERK then activates eIF2*α* and induces high expression of CHOP, which promotes apoptosis. Based on the western blot results (Figures 3(a), 3(e), 3(g), and 3(h)), OGD/R increased the level of phosphorylated eIF2*α* and further contributed to increasing the level of CHOP. Exposure of HT22 cells to *γ*-GC decreased the levels of p-eIF2*α* and CHOP. The IRE1*α*-TRAF2-JNK axis is another way to induce ER stress-mediated apoptosis. The interaction between IRE1*α* and TRAF2 causes the activation of JNK [27]. Here, we performed immunoprecipitation to determine the role of *γ*-GC in the IRE1*α*-TRAF2 interaction. As shown in Figure 3(f), the binding of TRAF2 to IRE1*α* was reinforced by OGD/R but disrupted by *γ*-GC. Consistent with the effect of *γ*-GC on the IRE1*α*-TRAF2 interaction, the activation of JNK caused by OGD/R was inhibited by the addition of *γ*-GC (Figures 3(g) and 3(h)). Overall, *γ*-GC exerted its neuroprotective effect by inhibiting ER stress-induced apoptosis via the PERK-eIF2*α*-CHOP and IRE1*α*-TRAF2-JNK axes.

### 3.4. *γ*-GC Protects Primary Cortical Neurons against OGD/R-Induced Death

**Figure 4:**
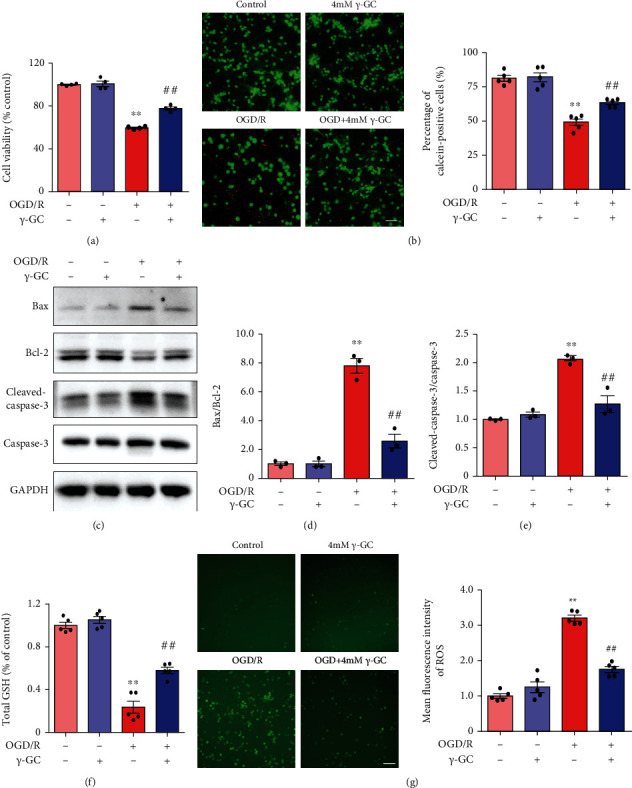
*γ*-GC protected primary cortical neurons against apoptosis and oxidative damage induced by OGD/R. Primary cortical neurons were pretreated with *γ*-GC for 1 hour and then subjected to 40 min of OGD followed by 12 h of reperfusion. (a) CCK-8 assays revealed cell viability. (b) Primary cortical neuronal apoptosis was examined by calcein/PI double staining. Scale bar: 50 *μ*m. The percentage of calcein-positive cells was calculated as calcein‐positive cells/(calcein‐positive cells + PI‐positive cells). Bax, Bcl-2, and cleaved caspase-3 were examined after 6 h of reperfusion. (c) Representative western blot images of Bax/Bcl-2 and cleaved caspase-3/caspase-3. (d, e) Quantification of Bax/Bcl-2 and cleaved caspase-3/caspase-3 levels. (f) The level of total GSH in primary cortical neurons. (g) The level of intracellular ROS was visualized and quantified as the mean fluorescence intensity. Scale bar: 100 *μ*m. All data are presented as the mean ± SEM. ^∗^*p* < 0.05, ^∗∗^*p* < 0.01 versus the control group. ^**#**^*p* < 0.05, ^**##**^*p* < 0.01 versus the OGD/R group.

The evidence showed that *γ*-GC mitigated ER stress-induced apoptosis to rescue HT22 cells from OGD/R insult, which prompted us to ascertain the effect of *γ*-GC on primary cortical neurons after OGD/R. A CCK-8 assay and calcein/PI labeling were used to examine the neuroprotective effect of *γ*-GC on primary neurons. The CCK-8 assay showed that *γ*-GC inhibited neuronal death induced by OGD/R (Figure 4(a)). *γ*-GC increased the percentage of calcein-labeled cells while decreasing the percentage of PI-labeled cells (Figure 4(b)), which was consistent with the CCK-8 assay results. Additionally, *γ*-GC-treated primary neurons were evaluated by western blot analysis to determine the ratio of Bax/Bcl-2 and the level of cleaved caspase-3 (Figures 4(c)–4(e)). We found that although OGD/R increased the ratio of Bax/Bcl-2 and the level of cleaved caspase-3, *γ*-GC abrogated this increase to some degree in primary neurons. Thereafter, the levels of total GSH and intracellular ROS were determined (Figures 4(f) and 4(g)). As predicted, OGD/R disturbed the balance between GSH and ROS in primary neurons. *γ*-GC increased the level of total GSH and scavenged intracellular ROS. Consistently, *γ*-GC alleviated excessive ROS generation and protected primary neurons from the OGD/R insult.

### 3.5. *γ*-GC Alleviates OGD/R-Induced ER Stress in Primary Cortical Neurons

**Figure 5:**
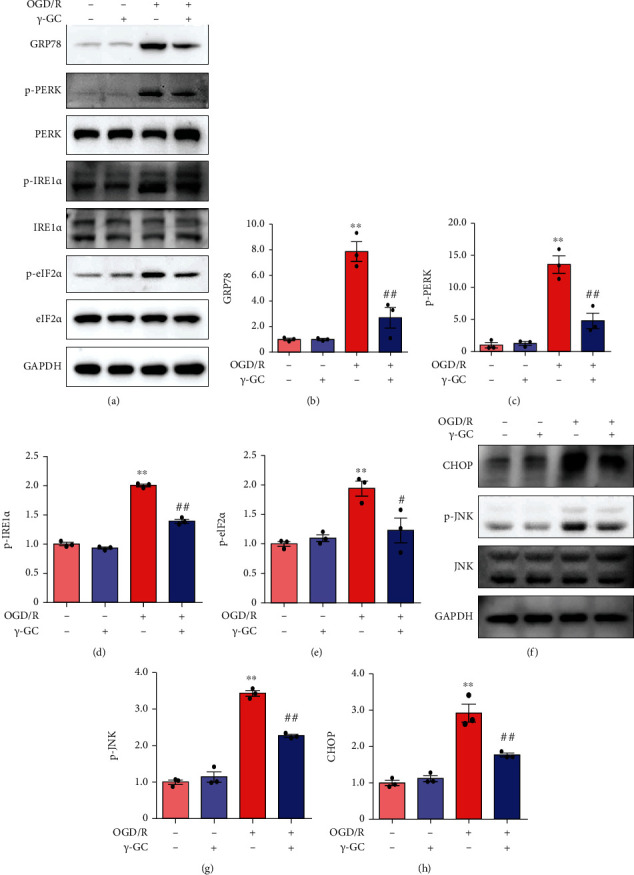
*γ*-GC exerted its neuroprotective effect by suppressing ER stress-induced apoptosis in primary neurons. (a) Western blot analysis of sensors of ER stress, including GRP78, p-PERK, p-IRE1*α*, and p-eIF2*α*, after primary cortical neurons were exposed to 1 h of reperfusion. (b–e) Quantification of GRP78, p-PERK/PERK, p-IRE1*α*/IRE1*α*, and p-eIF2*α*/eIF2*α* levels. (f) The levels of CHOP and p-JNK were measured by western blot analysis after 6 h of reperfusion. (g, h) Quantification of CHOP and p-JNK/JNK expression. All data are presented as the mean ± SEM. ^∗^*p* < 0.05, ^∗∗^*p* < 0.01 versus the control group. ^**#**^*p* < 0.05, ^**##**^*p* < 0.01 versus the OGD/R group.

Having confirmed that *γ*-GC was of value in ameliorating OGD/R-induced damage to primary cortical neurons, we performed western blot analysis to validate whether the PERK-eIF2*α*-CHOP and IRE1*α*-TRAF2-JNK pathways were still inhibited in *γ*-GC-treated primary neurons. Consistent with HT22 cells, primary neurons exhibited increased levels of GRP78 after OGD/R. However, exposure of primary neurons to *γ*-GC reversed this elevation (Figures 5(a) and 5(b)), indicating the suppression of ER stress. Likewise, after being subjected to OGD/R insult, primary neurons had increased levels of phosphorylated PERK and IRE1*α*, followed by the activation of eIF2*α* (Figures 5(a) and 5(c)–5(e)). However, *γ*-GC-treated primary neurons exhibited the opposite results: the activation of PERK and IRE1*α* was reduced, and subsequently, the level of phosphorylated eIF2*α* was decreased. Sequentially, OGD/R induced high levels of CHOP and JNK activation in primary neurons, indicating ER stress-induced apoptosis (Figures 5(f)–5(h)). After treatment with *γ*-GC, primary neurons had lower levels of CHOP and phosphorylated JNK. Similar to the effect of *γ*-GC on HT22 cells, *γ*-GC protected primary neurons against OGD/R by inhibiting ER stress-induced apoptosis.

### 3.6. *γ*-GC Protects against Ischemic Brain Injury in MCAO Mice

**Figure 6:**
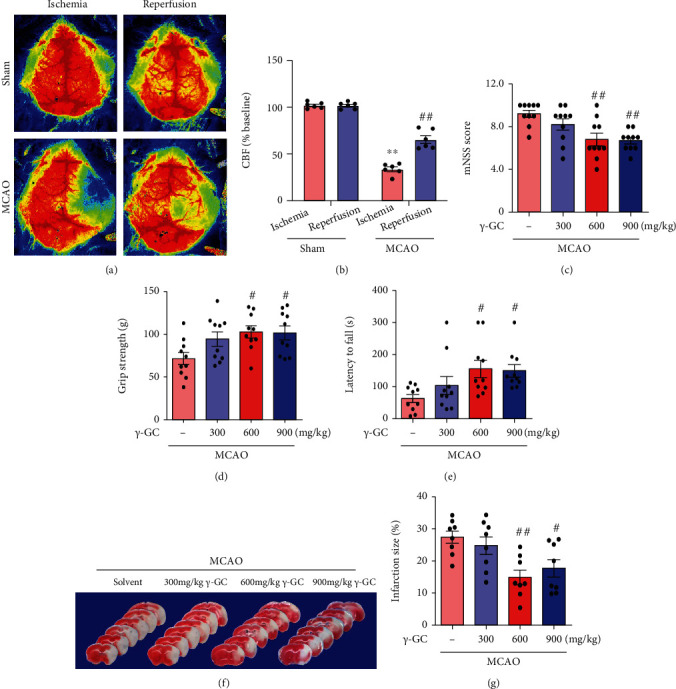
*γ*-GC alleviated neurological deficits in mice subjected to MCAO. 24 h after MCAO, the mice were sacrificed for in vivo examination. (a, b) Cerebral blood flow was measured by LSCI. Behavioral tests, including (c) mNSS, (d) grip strength, and (e) rotarod test, were performed to evaluate neurological function (*n* = 10). (f) The infarct area was visualized by TTC staining (*n* = 8). (g) Quantification of infarct volume. All data are presented as the mean ± SEM. ^∗^*p* < 0.05, ^∗∗^*p* < 0.01 versus the sham group. ^**#**^*p* < 0.05, ^**##**^*p* < 0.01 versus the MCAO group.

To investigate the neuroprotective role of *γ*-GC in ischemic stroke in vivo, we established mouse MCAO models. LSCI showed that cerebral blood flow was reduced to approximately 33.80 ± 2.466% when the mice were subjected to MCAO and restored to 65.41 ± 4.142% after reperfusion (Figures 6(a) and 6(b)). Thus, the mouse MCAO model successfully simulated the ischemia-reperfusion process. The mNSS, forepaw grip strength measurement, and rotarod test were conducted to observe the neurological outcomes of mice after MCAO (Figures 6(c)–6(e)). The administration of 600 or 900 mg/kg *γ*-GC significantly improved neurological deficits, which were reflected by decreases in the mNSS, improvements in grip strength, and the extension of time on the rotarod. Consistent with neurological recovery, the cerebral infarction size (Figures 6(f) and 6(g)) significantly shrank after the administration of *γ*-GC. These results confirmed that *γ*-GC could partially improve neurological deficit symptoms caused by ischemia/reperfusion damage. Although the dose of 300 mg/kg was not effective enough, 600 and 900 mg/kg *γ*-GC did work. A dose of 600 mg/kg was selected for the following experiment.

### 3.7. *γ*-GC Protects Ischemia-Induced Neurons by Inhibiting ROS Production and ER Stress

**Figure 7:**
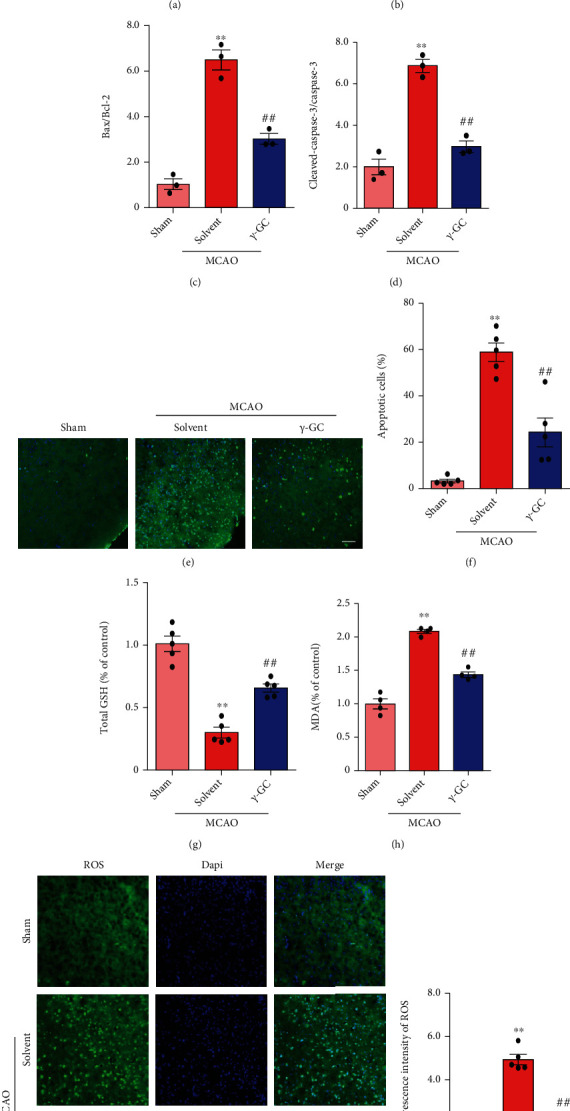
*γ*-GC protected MCAO mice against focal cerebral ischemic injury. (a) Image showing the ischemic penumbra that was isolated 24 h after MCAO for subsequent experiments. (b) Western blot analysis of apoptosis-associated proteins, including Bax, Bcl-2, and cleaved caspase-3. (c, d) The levels of Bax/Bcl-2 and cleaved caspase-3/caspase-3 were quantified. (e, f) Apoptotic cells were examined by TUNEL assays. Scale bar: 50 *μ*m. (g) Measurement of total GSH in the ischemic penumbra. (h) Evaluation of the MDA level in the ischemic penumbra. (i, j) Representative images and quantification of intracellular ROS levels in the ischemic penumbra. Scale bar: 50 *μ*m. All data are presented as the mean ± SEM. ^∗^*p* < 0.05, ^∗∗^*p* < 0.01 versus the sham group. ^**#**^*p* < 0.05, ^**##**^*p* < 0.01 versus the MCAO group.

Since *γ*-GC alleviated neurological deficits, further experiments were performed to investigate the protective effect of *γ*-GC in vivo. The ischemic penumbra was isolated for subsequent analysis (Figure 7(a)). Apoptosis-related proteins were examined by western blot analysis (Figures 7(b)–7(d)). As anticipated, ischemia/reperfusion elevated the ratio of Bax/Bcl-2 and the level of cleaved caspase-3. Consistent with the in vitro results, the oral administration of *γ*-GC decreased the ratio of Bax/Bcl-2 and the cleavage of caspase-3. Furthermore, apoptotic cells were examined by the TUNEL assay and are shown as TUNEL-positive cells. As shown in Figures 7(e) and 7(f), many TUNEL-positive cells were present in the brain slices after MCAO. However, the administration of *γ*-GC diminished the number of apoptotic cells. Thus, *γ*-GC helped to offset ischemia/reperfusion damage in brain tissue by inhibiting apoptosis. Since *γ*-GC reduced the level of ROS, which was confirmed in vitro, we continued to verify the antioxidant effect of *γ*-GC in vivo. As expected, ischemia/reperfusion injury consumed total GSH, increased the level of MDA, and increased the mean fluorescence intensity of ROS in brain tissue. The administration of *γ*-GC significantly increased the level of total GSH, and accordingly, the levels of MDA and ROS decreased (Figures 7(g)–7(j)).

**Figure 8:**
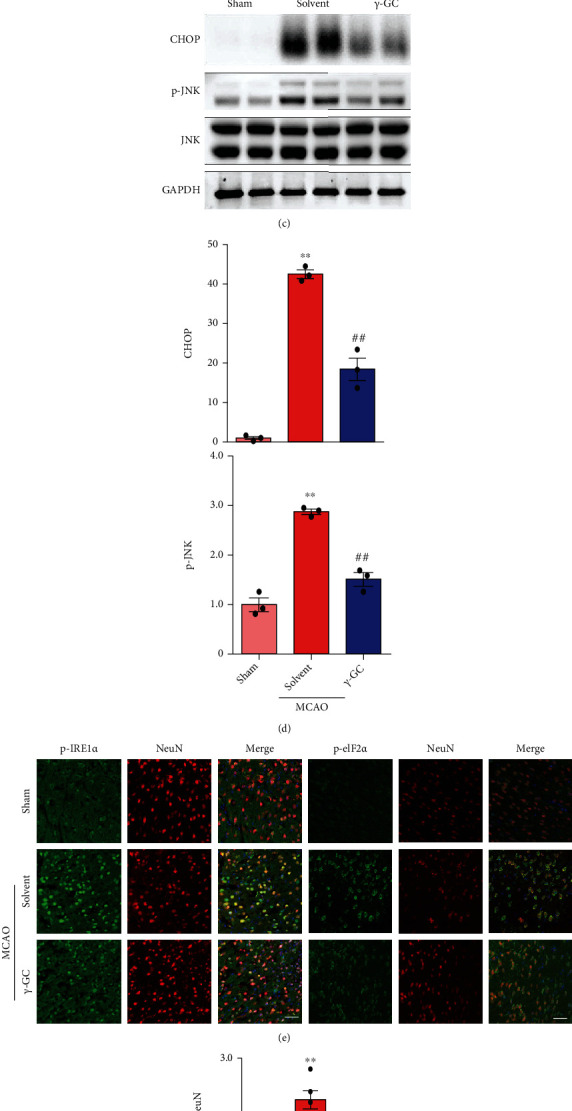
*γ*-GC exerted its neuroprotective effect in vivo by alleviating ER stress. (a) Western blot analysis of GRP78, p-PERK, p-IRE1*α*, and p-eIF2*α* in the sham, MCAO+solvent, and MCAO+*γ*-GC groups 24 h after modeling. (b) Quantification of GRP78, p-PERK/PERK, p-IRE1*α*/IRE1*α*, and p-eIF2*α*/eIF2*α* levels in the ischemic penumbra. (c, d) The levels of CHOP and p-JNK/JNK were determined by western blot analysis. (e) The changes in p-IRE1*α* and p-eIF2*α* were visualized by immunofluorescence staining. Scale bar: 50 *μ*m. (f) Quantification of p-IRE1*α*/NeuN and p-eIF2*α*/NeuN. All data are presented as the mean ± SEM. ^∗^*p* < 0.05, ^∗∗^*p* < 0.01 versus the sham group. ^**#**^*p* < 0.05, ^**##**^*p* < 0.01 versus the MCAO group.

The protective role of *γ*-GC in vivo was consistent with that in vitro. However, whether *γ*-GC protects brain tissue from ischemia/reperfusion injury by inhibiting ER stress-induced apoptosis was to be confirmed, since the internal environment is very complicated. According to the western blot results, ischemia/reperfusion damage markedly increased the expression levels of GRP78, p-PERK, p-IRE1*α*, and p-eIF2*α* in the penumbra, indicating the activation of the PERK and IRE1*α* axes (Figures 8(a) and 8(b)). The administration of *γ*-GC partially abrogated this elevation. Furthermore, MCAO mice treated with *γ*-GC had lower levels of CHOP and p-JNK in the penumbra than mice subjected to MCAO alone, indicating that *γ*-GC suppressed apoptosis caused by ER stress (Figures 8(c) and 8(d)). Immunofluorescence staining of neurons in the penumbra with anti-p-IRE1*α* and anti-p-eIF2*α* antibodies confirmed the protective effect of *γ*-GC against ER stress. As shown in Figures 8(e) and 8(f), the fluorescence of p-IRE1*α* and p-eIF2*α* was markedly enhanced in NeuN-labeled cells in MCAO mouse brain tissue, whereas treatment with *γ*-GC inhibited the overactivation of IRE1*α* and eIF2*α*. Overall, both in vitro and in vivo, *γ*-GC protected against ischemia/reperfusion injury by alleviating apoptosis mediated by the PERK/CHOP and IRE1*α*/JNK pathways.

## 4. Discussion

**Figure 9:**
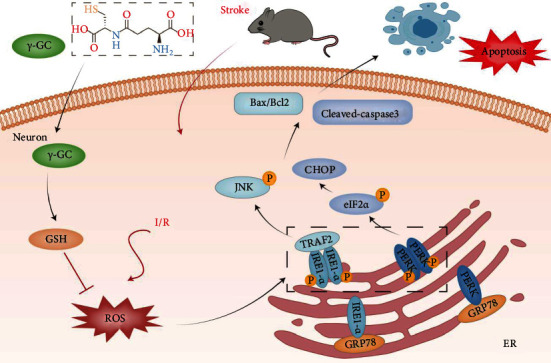
*γ*-GC protected against ischemia/reperfusion-induced neuronal apoptosis by inhibiting ROS-mediated ER stress.

Excessive ROS formation triggers neuronal apoptosis and is considered one of the key factors contributing to reperfusion injury. Rescuing neurons in the penumbra, which is between the normal region and ischemic core, is a potential strategy to improve ischemic brain injury. In the present study, we showed that the administration of exogenous *γ*-GC reduced HT22 neuronal cell apoptosis after OGD/R challenge by effectively increasing GSH and reducing ROS levels. The transcriptomics analysis results indicated that the underlying mechanism was involved in ER stress-induced apoptosis. These findings were verified in primary cortical neurons and in vivo. In mouse models of MCAO, *γ*-GC treatment reduced infarct volume and improved neurological and motor functions. Additionally, *γ*-GC administration mitigated oxidative stress, as suggested by increased GSH and decreased ROS and MDA levels. *γ*-GC attenuated penumbra neuronal apoptosis by inhibiting the activation of PERK and IRE1*α* in the ER stress signaling pathway in ischemic brain tissues. Overall, our results provide important evidence that *γ*-GC plays a neuroprotective role against ischemic injury in vivo and in vitro, and these effects are mediated by attenuating oxidative stress and subsequent ER stress-induced neuronal apoptosis. A mechanistic model of the protective effects of *γ*-GC is illustrated in Figure 9.

GSH is the most abundant thiol present in almost all cells and tissues, and it serves as an important antioxidant that can protect the cell from oxidative damage. Low plasma GSH levels are often seen in stroke patients [28]. Zhang et al. designed and synthesized two distinctive fluorescent probes to specifically observe the levels of GSH in living cells and in vivo during ischemia and reperfusion. Researchers found that low GSH levels were associated with cerebral infarction and the exacerbation of apoptosis in MCAO mice, and pretreatment with a GSH synthase inhibitor worsened ischemic brain damage [29]. GSH is a tripeptide composed of glutamate, cysteine, and glycine. Prior studies have shown that hypoxia induces a decrease in cellular GSH stores and concomitant inhibition of GSH biosynthesis, which correlates with impaired transport of the substrate cystine [30]. It was first confirmed in 1983 that *γ*-GC, an intermediate dipeptide with a sulfhydryl group in the GSH synthesis pathway, could increase the levels of GSH in the kidney [20]. Further studies have demonstrated that *γ*-GC and its esterified form GCEE can protect against oxidative damage in a variety of cell types through catalysis by GSS to produce intracellular GSH [23, 24, 31]. Recent studies have shown that compared with NAC and GSH supplementation, *γ*-GC administration was more effective in preventing lipopolysaccharide- (LPS-) or cecal ligation and puncture- (CLP-) induced systemic inflammatory responses [32]. These studies indicated that increasing GSH levels was beneficial to many diseases, such as ischemic stroke, and the best way to increase GSH in cells is to administer *γ*-GC. In this study, we showed for the first time that *γ*-GC treatment reduced neuronal apoptosis in models of brain ischemia in vitro and in vivo. Exogenous *γ*-GC elevated the levels of GSH in cultured neuron and ischemic brain tissues, accompanied by a decrease in oxidative damage.

To further address how *γ*-GC reduced neuronal apoptosis after inhibiting ROS, we examined the transcriptomes of cells after different treatments. Transcriptome analysis identified ER stress signaling pathways that were significantly altered by OGD/R in HT22 cells that were treated with *γ*-GC. Emerging evidence demonstrates that excessive ROS in a pathological state can trigger ER stress in vivo and in vitro [33]. Treatment with antioxidants protected old mice from oxidative stress-induced kidney injury and normalized ER stress responses [34]. Moreover, an ROS scavenger decreased caspase cascade-mediated apoptosis in human T cells and attenuated tissue injury during liver ischemia-reperfusion damage by blocking ER stress [35, 36]. ER stress is triggered by the accumulation of unfolded or misfolded proteins in the ER. Initially, the UPR is activated to induce adaptive programs to maintain homeostasis as much as possible. GRP78, PERK, and IRE1*α* are important parts of the UPR. If ER stress is prolonged, the UPR will induce apoptosis [37]. In ischemic stroke, ER stress plays a role in neuronal death. The accumulation of unfolded or misfolded proteins triggers excessive and persistent ER stress. Then, PERK and IRE1*α* are separated from GRP78 and autophosphorylated to activate downstream pathways and eventually induce apoptosis [38]. The activation of PERK will then phosphorylate eIF2*α* and ultimately induce the expression of CHOP. CHOP is an indicator of ER stress-induced apoptosis, and it has been validated that CHOP can upregulate the ratio of Bax/Bcl-2 and the cleavage of caspase-3 [39, 40]. Li et al. found that the activation of PERK-eIF2*α*-CHOP exacerbated ischemic stroke, while hairy and enhancer of split 1 (Hes1) exerted a protective effect by halting apoptosis induced by the PERK-CHOP pathway [41]. The IRE1*α* pathway is another mediator of apoptosis. The interaction between IRE1*α* and TRAF2 activates the JNK pathway and ultimately induces apoptosis. Therefore, a phosphodiesterase 4 (PDE4) inhibitor can suppress apoptosis in ischemic stroke by interrupting the binding of IRE1*α* and TRAF2 [42]. These studies are consistent with our work. We explored various in vitro and in vivo ischemia/reperfusion models and confirmed that *γ*-GC reduced ischemic stroke injury by inhibiting apoptosis via the PERK-eIF2*α*-CHOP and IRE1*α*-TRAF2-JNK axes.

In the in vivo experiment, the *γ*-GC concentration we used was taken into consideration based on previous studies. A randomized human trial confirmed that oral administration of 2 g or 4 g of *γ*-GC could effectively increase lymphocyte GSH levels [21]. The dose conversion from humans to mice is multiplied by 12 [43]. Therefore, we planned to assess *γ*-GC concentrations of 300 mg/kg and 600 mg/kg. It has been reported that 1200 mg/kg *γ*-GC reduced sepsis lethality and attenuated systemic inflammatory responses in mice with no toxicity or side effects [32]. Therefore, we also designed a high *γ*-GC group that was treated with 900 mg/kg. Our data showed that both 600 mg/kg and 900 mg/kg *γ*-GC significantly improved neurological deficits. Overall, a *γ*-GC concentration of 600 mg/kg was selected for the following experiments.

In our work, we confirmed that *γ*-GC administration increased the level of GSH, which could relieve oxidative stress. Quintana-Cabrera et al. found that *γ*-GC scavenged ROS regardless of GSH concentration [44], which is a complement to our work. Namely, *γ*-GC can alleviate oxidative injury by itself and by increasing the level of GSH. However, in ischemia/reperfusion injury, whether *γ*-GC can exert its antioxidant effect independently of GSH has not been investigated. Therefore, this aspect awaits further study in our subsequent work.

Apart from ER stress, RNA-seq also suggested that the mechanism by which *γ*-GC alleviates ischemic stroke is associated with ferroptosis and the TNF signaling pathway. Ferroptosis is a form of programmed cell death that is distinct from apoptosis and is highly related to oxidized lipid species. In addition, ferroptosis is a therapeutic target to inhibit neuronal death in stroke [45]. Moreover, inflammation is a principal contributor to ischemic injury. *γ*-GC exerts an anti-inflammatory effect on AD and sepsis [23, 32]. Therefore, in a future study, we can further explore whether *γ*-GC has the capacity to combat inflammation and inhibit ferroptosis.

## 5. Conclusion

In summary, *γ*-GC exerted its neuroprotective effect in vitro and in vivo. As an immediate precursor of GSH, *γ*-GC significantly increased the level of GSH and scavenged intracellular ROS. Furthermore, transcriptome analysis suggested that *γ*-GC relieved ER stress in ischemic stroke, and we confirmed that *γ*-GC inhibited neuronal apoptosis mediated by the PERK-eIF2*α*-CHOP and IRE1*α*-TRAF2-JNK axes. Hence, the application of *γ*-GC in the treatment of cerebral ischemia has a promising future.

## Data Availability

All data are available upon request.
